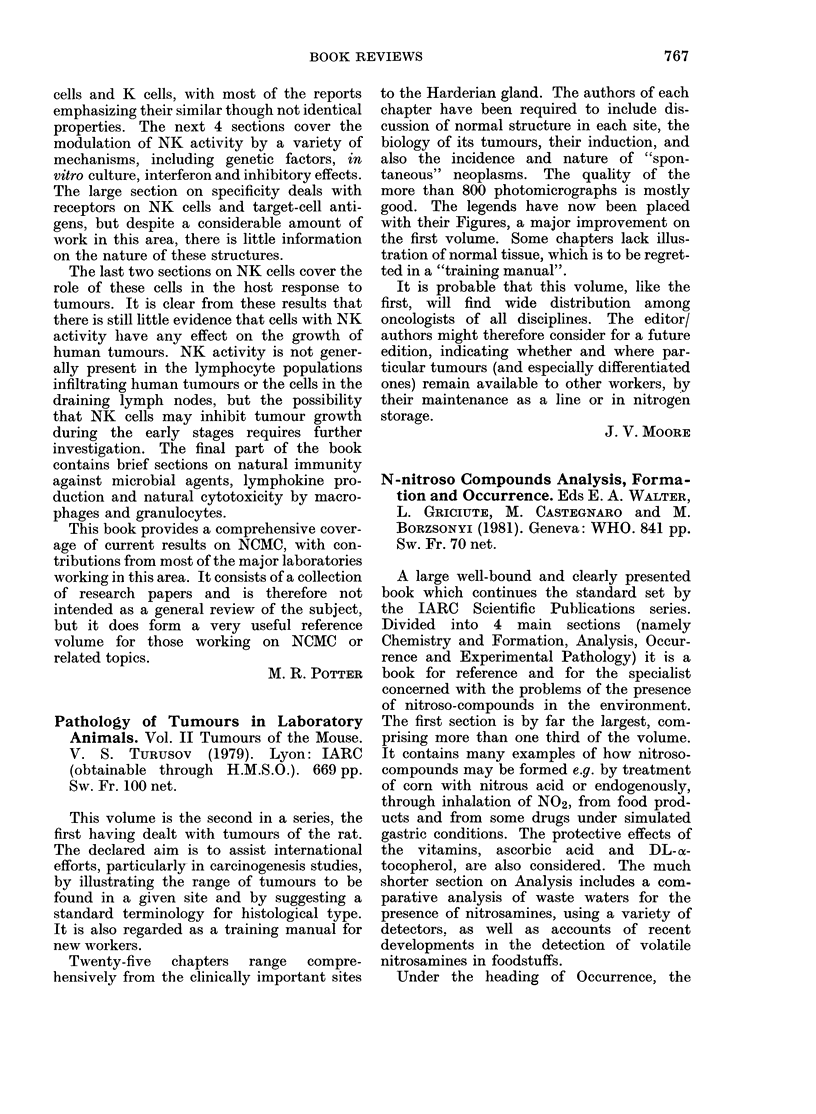# Pathology of Tumours in Laboratory Animals

**Published:** 1981-11

**Authors:** J. V. Moore


**Pathology of Tumours in Laboratory Animals.** Vol. II Tumours of the Mouse. V. S. TURUSOV (1979). Lyon: IARC (obtainable through H.M.S.O.). 669 pp. Sw. Fr. 100 net.

This volume is the second in a series, the first having dealt with tumours of the rat. The declared aim is to assist international efforts, particularly in carcinogenesis studies, by illustrating the range of tumours to be found in a given site and by suggesting a standard terminology for histological type. It is also regarded as a training manual for new workers.

Twenty-five chapters range comprehensively from the clinically important sites to the Harderian gland. The authors of each chapter have been required to include discussion of normal structure in each site, the biology of its tumours, their induction, and also the incidence and nature of "spontaneous" neoplasms. The quality of the more than 800 photomicrographs is mostly good. The legends have now been placed with their Figures, a major improvement on the first volume. Some chapters lack illustration of normal tissue, which is to be regretted in a "training manual".

It is probable that this volume, like the first, will find wide distribution among oncologists of all disciplines. The editor/authors might therefore consider for a future edition, indicating whether and where particular tumours (and especially differentiated ones) remain available to other workers, by their maintenance as a line or in nitrogen storage.

J. V. MOORE